# Hippocampal volume moderates the association between cerebrospinal fluid growth-associated protein 43 and episodic memory performance in older adults

**DOI:** 10.1080/13825585.2025.2562203

**Published:** 2025-09-24

**Authors:** Ann J. Lee, Olivia Horn, Scott M. Hayes

**Affiliations:** a Department of Psychology, The Ohio State University, Columbus, OH, USA; b Chronic Brain Injury Initiative, The Ohio State University, Columbus, OH, USA

**Keywords:** Alzheimer’s disease, aging, biomarkers, episodic memory, magnetic resonance imaging

## Abstract

This study examined the relationships between fluid and imaging biomarkers associated with memory (cerebrospinal fluid growth-associated protein 43 [CSF GAP-43] and hippocampal volume) and episodic memory performance in 586 older adults (mean age = 72.6 years, SD = 7.2) from the Alzheimer’s Disease Neuroimaging Initiative who completed CSF collection, Magnetic Resonance Imaging, and neuropsychological testing. Hierarchical linear regressions assessed whether FreeSurfer-derived total hippocampal volume or hippocampal subfield volume (subiculum, presubiculum, dentate gyrus, CA1) moderated the association between GAP-43 and episodic memory performance. GAP-43 and hippocampal volume were independently associated with episodic memory performance. Greater GAP-43 was associated with lower episodic memory performance in older adults with average or below-average (−1 SD) total and subfield (subiculum, presubiculum, dentate gyrus) hippocampal volume. No associations were observed in participants with above-average (+1 SD) volume. These findings suggest that GAP-43 may serve as a biomarker correlate of episodic memory impairment in those with average or below-average hippocampal volume.

## Introduction

Alzheimer’s disease (AD) is a progressive neurodegenerative disease characterized by episodic memory deficits, brain gray matter atrophy, and pathological features such as amyloid-β plaques, hyperphosphorylated tau, and neuronal dysfunction (Aisen et al., 2017; Jack et al., 2024). The degeneration of synapses is an early key component of neurodegeneration in AD and is closely related to cognitive impairment (Holahan et al., 2007; Masliah et al., 2001; Terry et al., 1991), suggesting biomarkers of synaptic integrity as potential *in vivo* correlates of cognitive dysfunction.

Cerebrospinal fluid growth-associated protein 43 (CSF GAP-43) is a presynaptic protein essential for neuronal development as well as hippocampal and memory functions (Camporesi et al., 2020; Neve et al., 1988). Although previous work in mouse models has associated the overexpression of the GAP-43 protein specifically with memory dysfunction (Holahan et al., 2007), the majority of extant literature in humans has related increased CSF GAP-43 with worse global cognition (Lu, 2022; Öhrfelt et al., 2022; Qiang et al., 2022; Sandelius et al., 2019). It is important to assess whether the relationship between CSF GAP-43 and cognitive performance is domain specific, as deficits in specific cognitive domains are differentially associated with AD risk (Espinosa et al., 2013; Yaffe et al., 2006). Our previous work using the Alzheimer’s Disease Neuroimaging Initiative (ADNI) demonstrated a negative relationship between CSF GAP-43 and episodic memory performance, but not executive function performance, independent of other biomarkers of neuronal dysfunction (Lee et al., 2025). Although our previous study examined the contributions of CSF GAP-43 to domain-specific cognitive performance relative to other fluid biomarkers, it did not assess other measures, such as gray matter volume, that may explain additional variability in episodic memory performance beyond the variance accounted for by CSF GAP-43. Given that pathophysiological alterations in AD rarely occur in isolation (Jack et al., 2024; Masliah et al., 2001), it is important to examine multimodal indicators of pathology (e.g., fluid biomarkers, Magnetic Resonance Imaging [MRI]) that may contribute to hallmark domain-specific cognitive deficits in AD, such as episodic memory.

Episodic memory impairment is associated with reduced total hippocampal volume, including disproportionate reductions in older adults with AD (Opitz, 2014; Zilioli et al., 2024). In addition to an aggregate measure of hippocampal volume, prior work has shown that histologically distinct subfields of the hippocampal complex have regionally specific associations with cognitive functions (O’Shea et al., 2022; Zammit et al., 2017; Zilioli et al., 2024). Other work has demonstrated a negative relationship between total hippocampal volume and CSF GAP-43 (Lu, 2022; Qiang et al., 2022); however, these studies did not assess domain-specific cognitive performance, interaction effects between GAP-43 and hippocampal volume on cognitive performance, or regional specificity within the hippocampus, such as hippocampal subfield volume. Despite documented associations between hippocampal volume and episodic memory functions (O’Shea et al., 2022; Zammit et al., 2017; Zilioli et al., 2024) and between hippocampal volume and CSF GAP-43 (Lu, 2022; Qiang et al., 2022), few studies have examined whether CSF GAP-43 and hippocampal atrophy have additive and/or interactive effects on episodic memory performance. A previous study reported that CSF synaptosomal-associated protein 25 (SNAP-25), another biomarker of presynaptic dysfunction (Camporesi et al., 2020), moderated the association between axonal injury and temporoparietal gray matter (Saloner et al., 2022), suggesting that differential levels of synaptic dysfunction may be a pathogenic driver of atrophy in AD-related brain regions; however, that study did not assess cognitive outcomes.

To address these gaps in the literature, the primary goal of the current cross-sectional study was to examine whether CSF GAP-43 and hippocampal volume accounted for unique variance in episodic memory performance. Additional analyses examined whether the relationship between CSF GAP-43 and episodic memory performance was moderated by hippocampal volume, and exploratory analyses examined evidence of regional specificity among hippocampal subfields. Given that CSF GAP-43 and hippocampal volume reflect differing pathological mechanisms, our approach to examine GAP-43 and hippocampal volume as simultaneous predictors of episodic memory performance allowed for the assessment of synaptic and neurodegenerative pathologies and their independent and/or interactive effects on domain-specific cognitive performance. We hypothesized that greater CSF GAP-43 and lower total hippocampal volume would have additive effects on episodic memory. In addition, we explored whether any of the hippocampal subfields were differentially sensitive to episodic memory performance (O’Shea et al., 2022; Zammit et al., 2017; Zilioli et al., 2024). Finally, we hypothesized that the negative association between CSF GAP-43 and episodic memory performance would be stronger in older adults with below-average hippocampal volume compared to those with greater volumetric measures.

## Materials and methods

Data for this study were obtained from the ADNI database (adni.loni.usc.edu). ADNI was launched in 2003 as a public-private partnership, led by Principal Investigator Michael W. Weiner, MD. The primary goal of ADNI has been to test whether serial MRI, positron emission tomography (PET), biological markers, and clinical and neuropsychological assessment can be combined to measure the progression of mild cognitive impairment (MCI) and early AD. Extensions of the ADNI project were developed with aims to assess biomarkers at earlier stages of AD. Study procedures were approved by the Institutional Review Boards of participating institutions and informed written consent was obtained from all participants at each site.

### Participants

Participants were selected from ADNI-GO and ADNI-2 based on available baseline CSF GAP-43, hippocampal volume, and composite episodic memory data. The final analysis sample included 586 participants aged 55 to 90 years (mean age = 72.6; mean education = 16.1 years; 46% female; Table 1). ADNI exclusion criteria included, but were not limited to, any significant neurologic disease other than AD and known structural brain abnormalities. The full inclusion/exclusion criteria are located in the ADNI procedures manual (https://adni.loni.usc.edu/help-faqs/adni-documentation/).

### CSF GAP-43

CSF GAP-43 was measured using an in-house enzyme-linked immunosorbent assay (ELISA) method at the Clinical Neurochemistry Laboratory at the University of Gothenburg, Sweden. Biomarker analyses were validated by ADNI and details are described elsewhere (Sandelius et al., 2019).

### Hippocampal volume

Participants completed whole-brain MRI on a 3-Tesla scanner following a standardized protocol and quality control (Jack et al., 2015). FreeSurfer-derived hippocampal volume data were acquired from the ADNI database. The ADNI FreeSurfer protocol included data pre-processing completed by Mayo Clinic and cortical reconstruction and volumetric segmentation conducted using the FreeSurfer image analysis suite (Fischl, 2012). In brief, the ADNI FreeSurfer protocol included motion correction, removal of non-brain tissue using a hybrid watershed/surface deformation procedure, Talairach transformation, intensity normalization, topology correction, and segmentation of subcortical white matter and deep gray matter volumetric structures (e.g., hippocampus). Each scan was segmented according to an atlas defined by FreeSurfer, allowing for comparison between groups at a single time point (Fischl & Dale, 2000). For additional details regarding FreeSurfer, see https://surfer.nmr.mgh.harvard.edu/fswiki/FreeSurferMethodsCitation. The ADNI quality control (QC) protocol for FreeSurfer segmentation involved inter-rater reliability between ADNI sites and implementation of a thorough visual QC rating system to exclude failed data processing, including global failure of segmentation estimation and misestimation of the hippocampus. For further details on the ADNI MRI protocol and FreeSurfer methods and QC, see ADNI’s website (https://adni.loni.usc.edu/help-faqs/adni-documentation/) and the associated methods paper (UCSF_FreeSurfer_Methods_and_QC_OFFICIAL.pdf). Participants who passed overall (“OVERALLQC”), right hippocampal subfield, and left hippocampal subfield QC were included in the current study to ensure QC of hippocampal ROIs. Participants with missing right and/or left hippocampal subfield QC ratings were excluded from the final analysis sample.

Total hippocampal volume was included as an aggregate volumetric measure of hippocampal gray matter integrity. In addition, four hippocampal regions-of interest (ROI; subiculum, presubiculum, dentate gyrus, Cornu Ammonis-1 [CA1]) were examined to assess potential region-specific associations. ROIs were selected based on extant literature on hippocampal subfields and episodic memory performance (Aslaksen et al., 2018; Broadhouse et al., 2019; O’Shea et al., 2022; Zammit et al., 2017; Zhang et al., 2023; Zhao et al., 2019; Zheng et al., 2018) and data availability. For instance, subiculum (O’Shea et al., 2022; Zammit et al., 2017; Zhao et al., 2019; Zheng et al., 2018), presubiculum (O’Shea et al., 2022; Zheng et al., 2018), dentate gyrus (Aslaksen et al., 2018; Broadhouse et al., 2019; O’Shea et al., 2022), and CA1 volume (Aslaksen et al., 2018; Zammit et al., 2017; Zheng et al., 2018) have been associated with tasks of episodic memory performance across multiple studies. Atrophy in CA1, subiculum, and presubiculum hippocampal subfields have also been implicated as early neuroanatomical markers of AD (Carlesimo et al., 2015; de Flores et al., 2015). Right and left hemisphere volumes were averaged and corrected for intracranial volume.

### Episodic memory assessment

Participants completed a neuropsychological assessment at the baseline visit. We used a composite score of episodic memory (Rey Auditory Verbal Learning Test, the cognitive subscale of the Alzheimer’s Disease Assessment Scale, Logical Memory I and II, three word recall items from the Mini-Mental State Examination), validated in the ADNI cohort (Crane et al., 2012). For further details on the composite score, see the associated methods paper: ADNI_Methods_UWNPSYCHSUM.pdf.

### Control variables

Models controlled for age, sex, years of education, *APOE*-ɛ4 status, and Amyloid-β/Tau/Neurodegeneration (ATN) group. *APOE*-ɛ4 status was coded as either no *APOE*-ɛ4 allele or at least one *APOE*-ɛ4 allele. We controlled for ATN group status given that greater severity in AD proteinopathies (amyloid-β [Aβ_42_], phosphorylated tau [p-tau; 181P]) are associated with cognitive impairment (Delmotte et al., 2021; Jack et al., 2018). Pathological ATN profiles were determined by applying published cut-off values to each biomarker (CSF Aβ_42_ [A+]: < 977 pg/mL; CSF p-tau [T+]: > 27 pg/mL; neurodegeneration [N+]: ^18^F-fluorodeoxyglucose-positron emission composite ROI < 1.21 (Blennow et al., 2019; Jagust et al., 2009). Three ATN groups were created: 1) no AD pathology (A-T-N-), 2) suspected non-AD pathophysiology (A-T±N+ or A-T+N±), and 3) AD continuum (A+T±N±). Age and years of education were continuous covariates. Sex, *APOE*-ɛ4 status, and ATN group were categorical covariates with male, no *APOE*-ε4 allele, and no AD pathology group coded as reference categories.

### Statistical analysis

Statistical analyses were conducted using R (version 4.1.1717). All data were downloaded from the ADNI database (http://adni.loni.usc.edu/). Normality assumptions for all variables were inspected using Q-Q plots, and CSF GAP-43 was logarithmically transformed to approximate a normal distribution. Participants with missing data or data ± 3 standard deviations from the mean were removed from the final sample. All variables were standardized prior to analyses. The alpha level for tests was set as *p* < 0.05.

Hierarchical linear regressions were employed to examine whether CSF GAP-43 and hippocampal volume accounted for unique variance in episodic memory performance, and whether the association between CSF GAP-43 and episodic memory performance differed by total hippocampal volume or hippocampal subfield volume. Five separate models were run with one ROI as an independent variable to assess the main effect of each respective ROI on episodic memory performance. In each model, control variables (Step 1), CSF GAP-43 (Step 2), and the respective hippocampal ROI (Step 3) were added in separate steps to examine the additive and cumulative effect of predictor variables on episodic memory performance. At Step 3 of each model, episodic memory performance was regressed on a total of seven predictor variables (five control variables, CSF GAP-43, and one hippocampal ROI). To test for potential interaction effects, the CSF GAP-43 × Hippocampal ROI interaction was added in the final step of each model (Step 4). Significant interaction effects were probed using the *emmeans* package in R (Lenth, 2016). Confidence intervals (CI) indicate 95% CI.

## Results

### Study sample characteristics

Participant characteristics are presented in Table 1. 586 older adults were included in the final analysis sample. The mean age was 72.6 years (SD = 7.2), and the mean educational attainment was 16.1 years (SD = 2.7). 268 participants (46% of sample) were female and 268 (46% of sample) had at least one *APOE*-ɛ4 allele. Participants had a mean Mini-Mental State Examination score of 27.7 (SD = 2.5) and episodic memory composite score of 0.41 (SD = 0.9; Table 1).

### Associations between CSF GAP-43 and episodic memory performance by hippocampal volume across participants

**Figure 1.**
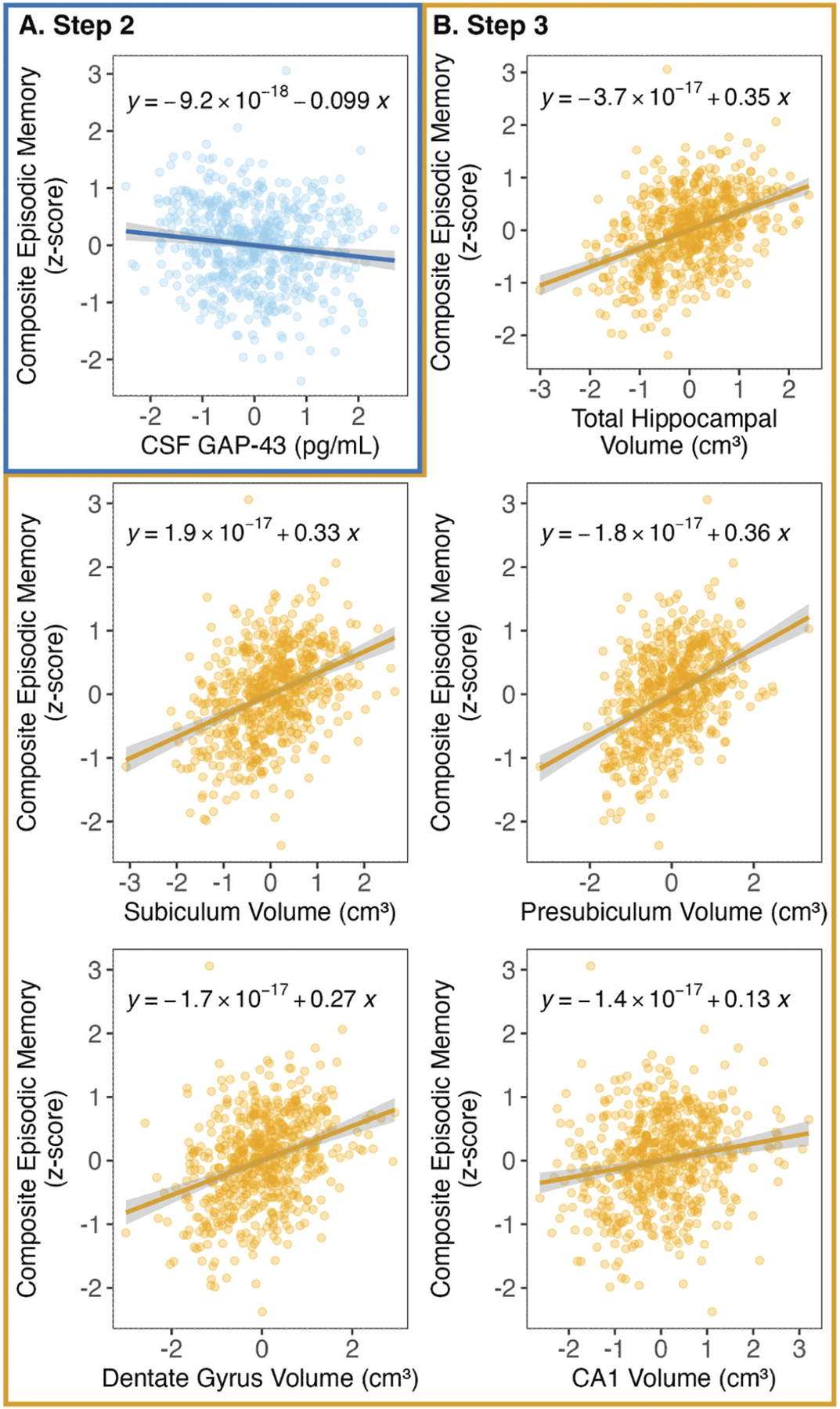
Main effects of CSF GAP-43 and hippocampal ROI volumes on episodic memory performance. (A) In Step 2 of the hierarchical regression model (after adjusting for age, sex, years of education, *APOE*-ɛ4 status, and ATN group), CSF GAP-43 was negatively associated with episodic memory performance. (B) In Step 3, total hippocampal volume, subiculum volume, presubiculum volume, dentate gyrus volume, and CA1 volume were positively associated with episodic memory performance. Regression equations for residuals are shown for each plot. Abbreviations: ATN = amyloid-β/tau/neurodegeneration; CA1 = Cornu Ammonis-1; CSF = cerebrospinal fluid, GAP-43 = growth-associated protein 43.

We examined the relationship between CSF GAP-43 and episodic memory performance and whether associations were moderated by total or subfield hippocampal volume. In Step 1 of all models, control variables (age, sex, years of education, *APOE*-ɛ4 status, ATN group) accounted for 33.67% of the variance in episodic memory performance (Model *F*[6,579] = 48.99, *p* < 0.001). In Step 2 of all models, CSF GAP-43 was added as an independent regressor to examine the main effect of GAP-43 on episodic memory after adjusting for control variables. Across all models, adding CSF GAP-43 accounted for an additional 1.15% of the variance in episodic memory performance (Model *F*[7,578] = 44.12, *p*s < 0.001; total R^2^ = 0.35) and improved each model (△R^2^: *p*s < 0.01). At Step 2 (after adjusting for control variables), CSF GAP-43 was negatively associated with episodic memory performance ($\hat{\beta }$ = −0.10; *p*s < 0.01; Figure 1A).

In Step 3 of all models, the respective hippocampal ROI (total hippocampal volume, subiculum, presubiculum, dentate gyrus, or CA1 volume) was added to each model. All five ROIs were positively associated with episodic memory performance (all Model *F p*s: < 0.001) and improved their respective models (△R^2^: *p*s < 0.001; Figure 1B). Total hippocampal volume accounted for an additional 11.09% of the variance in episodic memory performance (*F*[8,577] = 61.23; total R^2^ = 0.46). Subiculum volume accounted for an additional 9.75% of the variance in episodic memory (*F*[8,577] = 58.00; total R^2^ = 0.45). Presubiculum volume accounted for an additional 11.97% (*F*[8,577] = 63.43; total R^2^ = 0.47). Dentate gyrus volume accounted for an additional 7.46% (*F*[8,577] = 52.84; total R^2^ = 0.42). CA1 volume accounted for an additional 1.82% (Model *F*[8,577] = 41.71; total R^2^ = 0.37). Notably, at Step 3 (after adding each respective hippocampal ROI), CSF GAP-43 remained negatively associated with episodic memory performance in all models ($\hat{\beta }$s = −0.09 to −0.08, *p*s < 0.01), suggesting that hippocampal ROIs and CSF GAP-43 were independently associated with episodic memory performance.

**Figure 2.**
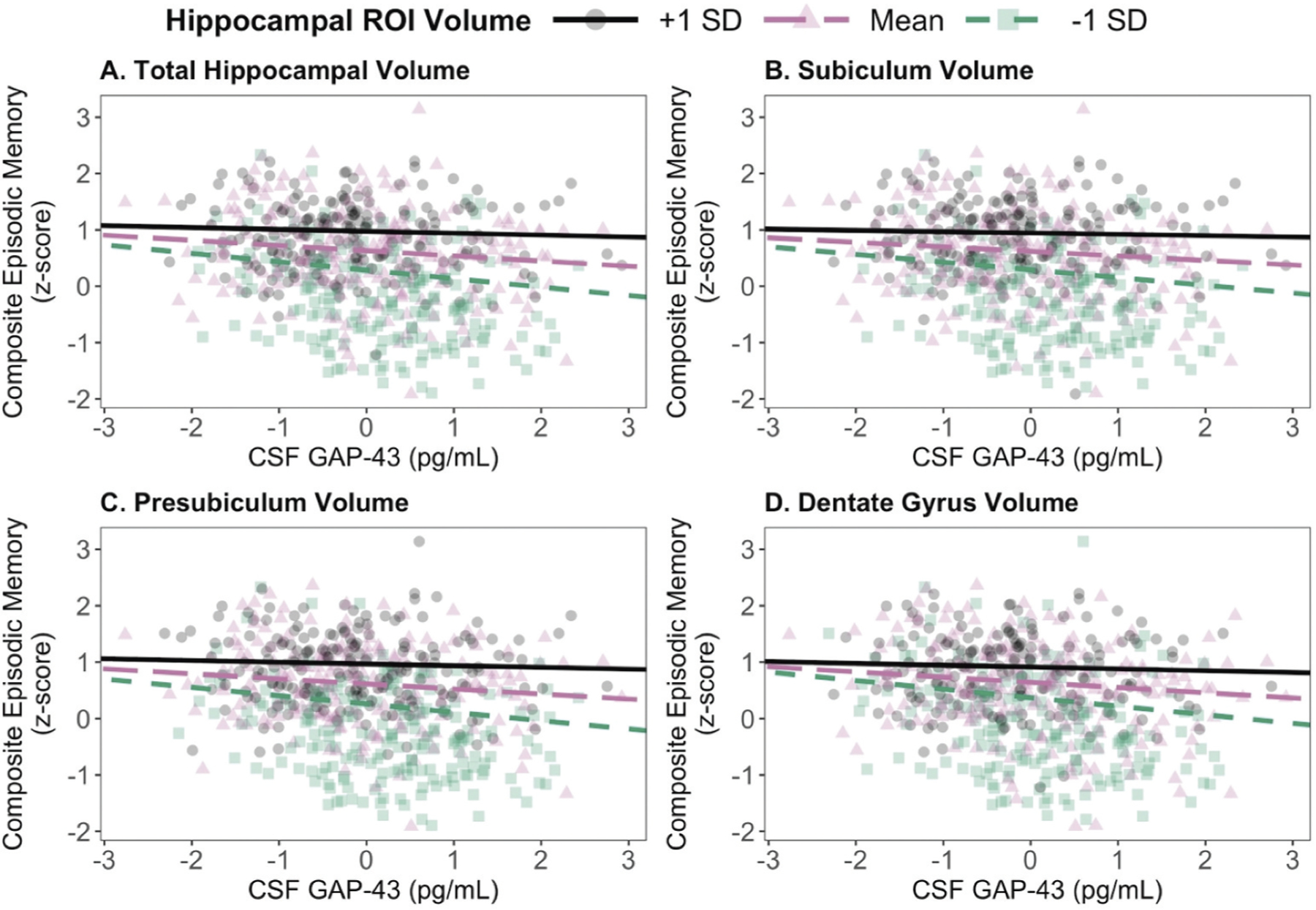
Associations between CSF GAP-43 and episodic memory performance with hippocampal ROI volume as the moderating variable. Significant interactions with episodic memory were observed between CSF GAP-43 and (A) total hippocampal volume, (B) subiculum volume, (C) presubiculum volume, and (D) dentate gyrus volume on episodic memory performance. Post-hoc results showed that greater CSF GAP-43 was associated with worse episodic memory performance in older adults with average or below-average (−1 SD) total hippocampal volume and hippocampal subfield volumes. In contrast, no associations were observed between CSF GAP-43 and episodic memory performance in those with above-average (+1 SD) total hippocampal volume or hippocampal subfield volumes. Dashed colored lines indicate a significant slope; solid black lines indicate a non-significant slope. Abbreviations: CSF = cerebrospinal fluid, GAP-43 = growth-associated protein 43, ROI = region-of-interest, SD = standard deviation.

Lastly, in Step 4, the CSF GAP-43 × Hippocampal ROI interaction term was entered in each respective model. Significant interaction effects were observed for four of the five hippocampal ROIs (total hippocampal volume, subiculum, presubiculum, and dentate gyrus volume), such that the relationship between CSF GAP-43 and episodic memory performance was more negative in those with lower hippocampal volume (Table 2; Figure 2). The GAP-43 × CA1 volume interaction was not significant.

Post-hoc simple slopes analyses revealed that greater CSF GAP-43 was associated with worse episodic memory performance in participants with average or below-average (−1 SD) total and subfield hippocampal volume. Specifically, there was a negative association between CSF GAP-43 and episodic memory performance in older adults with below-average volume for the four previously mentioned ROIs: total hippocampus ($\hat{\beta }$ = −0.15, CI [−0.23, −0.07]), subiculum ($\hat{\beta }$ = −0.14, CI[−0.22, −0.06]), presubiculum ($\hat{\beta }$ = −0.15, CI[−0.23, −0.07]), and dentate gyrus ($\hat{\beta }$ = −0.15, CI[−0.23, −0.07]). There was also a negative association between CSF GAP-43 and episodic memory performance in older adults with average volume for the four ROIs: total hippocampus ($\hat{\beta }$ = −0.09, CI[−0.15, −0.04]), subiculum($\hat{\beta }$ = −0.08, CI[−0.14, −0.02]), presubiculum ($\hat{\beta }$ = −0.09, CI[−0.15, −0.03]), and dentate gyrus ($\hat{\beta }$ = −0.09, CI[−0.15, −0.03]). In contrast, the association between CSF GAP-43 and episodic memory performance was null in older adults with above-average (+1 SD) total and subfield hippocampal volume. Pairwise differences of the simple slopes indicated that all slopes were significantly different from one another across all models (*p*s < 0.05).

To examine whether the observed CSF GAP-43 × Hippocampal ROI interaction was a general mechanism of neurodegeneration or present in specific ATN groups, exploratory analyses examined the associations between CSF GAP-43, hippocampal ROI volumes, and episodic memory in ATN classified no AD pathology participants (*n* = 182) and AD continuum participants (*n* = 312). Analyses were not run for the suspected non-AD pathophysiology group due to an insufficiently powered sample size (*n* = 92). The link between CSF GAP-43 and episodic memory performance was strongest in those with established AD pathology (AD continuum group; $\hat{\beta }$ = −0.16, *p* < 0.001), whereas the relationship was not significant in those without AD pathology (*p* = 0.81). However, we did not observe interaction effects between CSF GAP-43 and hippocampal ROIs in ATN stratified groups or groups stratified by neurodegeneration. See Supplementary Figure 1 and Supplementary Figure 2 for scatterplots displaying interaction effects by ATN group.

## Discussion

The assessment of fluid biomarkers of comorbid neuropathologies may augment hallmark biological indicators of AD (e.g., amyloid-β and phosphorylated tau (Jack et al., 2024)) to provide mechanistic insight into the multifactorial disease process on cognitive outcomes. The current study examined whether CSF GAP-43, a biomarker of synaptic dysfunction that is densely expressed in the hippocampus (Neve et al., 1988), and hippocampal volume (total and subfield volume) were independently associated with episodic memory performance. Additional analyses explored whether hippocampal volume moderated the association between CSF GAP-43 and episodic memory in older adults. Results showed that CSF GAP-43 and hippocampal volume were independently associated with episodic memory performance after adjusting for demographic variables and ATN group status. The relationship between CSF GAP-43 and episodic memory performance also varied as a function of hippocampal ROI volume: in older adults with average or below-average (−1 SD) total hippocampal or subfield (subiculum, presubiculum, dentate gyrus) volume, greater CSF GAP-43 was associated with worse episodic memory performance. In contrast, no robust associations were observed in older adults with above-average (+1 SD) total hippocampal or subfield volume. These findings demonstrated that both CSF GAP-43 and hippocampal volume were associated with episodic memory performance and suggest the role of GAP-43 as a potential correlate of episodic memory in those with average or below-average hippocampal volume.

Our findings highlight synaptic dysfunction as a mechanism contributing to deficits in episodic memory performance. Previous independent studies have reported associations between CSF GAP-43 and global cognitive decline (Lu, 2022; Öhrfelt et al., 2022; Qiang et al., 2022; Sandelius et al., 2019) and episodic memory impairment (Lee et al., 2025) among cognitively normal, MCI, and AD samples. Other studies have demonstrated associations between CSF GAP-43 and total hippocampal atrophy (Lu, 2022; Qiang et al., 2022). Postmortem neuropathological studies have also demonstrated increased regional CSF GAP-43 concentration and synaptic loss in the hippocampus in patients with AD compared to age-matched controls (Bogdanovic et al., 2000; Rekart et al., 2004). By examining CSF GAP-43 and hippocampal volume as simultaneous predictors of cognition, we extend the literature by demonstrating that greater CSF GAP-43 and reduced hippocampal volume were independently associated with episodic memory performance, and that the relationship between GAP-43 and episodic memory was moderated by hippocampal volume.

Concordant with extant literature, regression beta coefficients from the current study indicated stronger associations between hippocampal volume and episodic memory performance compared to associations with CSF GAP-43. The hippocampus, a bilateral medial temporal lobe structure, is essential for the formation and retrieval of episodic memories (Opitz, 2014). Multiple studies have linked reduced total and subfield hippocampal volume with memory impairment in older adults (O’Shea et al., 2022; Zammit et al., 2017; Zilioli et al., 2024). For instance, among participants without dementia, subiculum volume was positively associated with verbal and visual episodic memory and was suggested to be involved in the retrieval of information (Zammit et al., 2017). Larger presubiculum, subiculum, and dentate gyrus volumes have also been associated with higher scores on a delayed episodic memory task among patients with MCI (O’Shea et al., 2022). Additionally, we note that CSF GAP-43 added a statistically significant, although modest, amount of variance in episodic memory after adjusting for control variables. It is well established that demographic variables, such as greater age, fewer years of educational attainment, and *APOE*-ε4 genotype, are risk factors for cognitive decline (Riedel et al., 2016; van Hooren et al., 2007). Other work has demonstrated associations between ATN status and cognitive performance in older adult samples (Delmotte et al., 2021; Ebenau et al., 2020). Amyloid-β and tau pathology also have documented associations with synaptic loss in AD (Fagiani et al., 2019; Pereira et al., 2020; Tai et al., 2012; Wu et al., 2010). Consistent with this evidence base, our findings revealed that ATN AD pathology group status was significantly associated with episodic memory performance and had the greatest regression beta values relative to other control variables. Taken together, our findings suggest that episodic memory performance may be partially explained by pathologies that co-occur in the AD process (e.g., synaptic dysfunction), implicating CSF GAP-43 as a biomarker that may complement well-established predictors of episodic memory performance (e.g., hippocampal volume) in older adults even after adjusting for ATN group status. Elevations in CSF GAP-43 have also been suggested to be specific to AD (Sandelius et al., 2019). Given that CSF GAP-43 in ADNI was obtained via lumbar puncture, future advancements in candidate blood-based biomarkers of presynaptic dysfunction may improve the clinical utility of these findings. The first study to validate AD-induced changes in synaptic protein biomarkers in plasma demonstrated that neuro-exosomal GAP-43 correlated with CSF GAP-43 and discriminated between controls, MCI, and AD patients (Jia et al., 2021), suggesting promising avenues for future research with blood-based biomarkers of synaptic dysfunction. Future work may also wish to consider controlling for pathological AD status to avoid misattributing the effects of GAP-43 on episodic memory performance.

Additionally, greater CSF GAP-43 was associated with worse episodic memory performance specifically in older adults with average or below-average (−1 SD) total and subfield (subiculum, presubiculum, dentate gyrus) hippocampal volume, which is consistent with previous work (Lu, 2022; O’Shea et al., 2022; Qiang et al., 2022). We extend existing literature by demonstrating that hippocampal volume interacted with CSF GAP-43. A previous study examining CSF SNAP-25 (another presynaptic protein (Camporesi et al., 2020)) found that CSF neurofilament light chain (biomarker of neurodegeneration potentially analogous to hippocampal volume (Jack et al., 2024; Jung & Damoiseaux, 2024)) was associated with temporoparietal atrophy at greater SNAP-25 levels but not at lower levels (Saloner et al., 2022), which is consistent with our results. In contrast, our findings showed that the relationship between CSF GAP-43 and episodic memory performance was null in those with above-average (+1 SD) total hippocampal volume and in multiple hippocampal subfield volumes. Our findings are relatively consistent with the concept of brain reserve (Stern, 2009), such that older adults with larger hippocampal volume may not exhibit memory deficits commensurate with underlying pathology, such as synaptic dysfunction. For instance, autopsy studies reported that among individuals with AD pathology, those who were cognitively unimpaired at death were more likely to have preserved synaptic proteins compared to those with synaptic failure, suggesting the role of cognitive resilience and synaptic dysfunction on cognition (Arnold et al., 2013).

The hypothetical pathological cascade of AD postulates an initial accumulation of extracellular amyloid-β, followed by tau aggregation and synaptic dysfunction, subsequently leading to downstream tau-mediated neurodegeneration and cognitive impairment (Jack et al., 2010; Therriault et al., 2024). Consistent with this notion, prior studies have reported increased CSF GAP-43 concentrations, as a proxy of synaptic dysfunction, in those with low amyloid burden (Milà-Alomà et al., 2021) as well as those with pathogenic levels of amyloid-β but without tau pathology (Pereira et al., 2020), suggesting early alterations of GAP-43 in the progression of AD. Another study demonstrated that baseline CSF GAP-43 was associated with subsequent global cognitive decline and total hippocampal atrophy (Qiang et al., 2022). Moreover, our finding that CSF GAP-43 was associated with worse episodic memory performance in those with average or below-average hippocampal volume may indicate a synergistic effect of co-existing synaptic pathologies and downstream structural atrophy on episodic memory. Importantly, our results add to existing literature by demonstrating that the association between CSF GAP-43 and episodic memory performance depended on an aggregate measure of hippocampal integrity, suggesting that total volume, rather than regionally specific subfields, may be an adequate measure to assess interactive effects between CSF GAP-43 and hippocampal volume. Given the challenges of hippocampal subfield segmentation, including variability in segmentation methods, ambiguity in delineating anatomical boundaries between subfields, and segmentation errors (Canada et al., 2024), the use of total hippocampal volume may be a more reliable measure and mitigate potential concerns about measurement validity.

Neuropathological studies have demonstrated that CA1 volume exhibits early and prominent atrophy and neuronal loss in AD (de Flores et al., 2015; Padurariu et al., 2012) and is preferentially affected by AD neuropathology, including neurofibrillary tangle accumulation (Braak & Braak, 1991). An association between CA1 volume and episodic memory was observed, although contrary to our hypotheses, we did not observe an interaction between CSF GAP-43 and CA1 volume, suggesting that CA1 volume may be a neuroanatomical correlate of episodic memory performance irrespective of levels of GAP-43. Multiple studies have demonstrated associations between CA1 morphometry and episodic memory performance across non-demented/healthy (Aslaksen et al., 2018; Zammit et al., 2017; Zheng et al., 2018) and amnestic MCI and AD (Broadhouse et al., 2019; Kerchner et al., 2012) participants with presumed varying degrees of synaptic loss. CA1 volume, and not other subfield volumes, has also been associated with verbal memory performance irrespective of retrieval mode (e.g., free recall or recognition) (Aumont et al., 2023), consistent with the verbal memory tasks comprising the ADNI composite score. Alternatively, our findings may reflect limitations in FreeSurfer segmentation. A systematic review and meta-analysis found that FreeSurfer-derived CA1 volume was not the most severely reduced hippocampal subfield among older adults with MCI and AD, contrary to the authors’ hypotheses (Zhang et al., 2023). Another study reported that FreeSurfer-derived presubiculum, subiculum, and dentate gyrus volumes were associated with verbal episodic memory decline whereas CA1 volume was only associated with global cognitive decline (Doran et al., 2023). Both studies attributed discrepancies in CA1 findings to difficulties in delineating its anatomical boundaries using FreeSurfer (de Flores et al., 2015; Doran et al., 2023; Zhang et al., 2023). Indeed, CA1 volume added less variance to episodic memory performance relative to other hippocampal ROIs in the current study. Nonetheless, our results replicated previous findings associating CA1 volume with episodic memory performance (Aslaksen et al., 2018; Zammit et al., 2017; Zheng et al., 2018) and, further, suggest that this association may be independent of pathological synaptic states, though caution is warranted given the aforementioned methodological limitations.

Limitations of the present study include a highly educated and demographically and racially/ethnically homogenous sample, which limits the generalizability of our findings, and a cross-sectional analysis, which precludes causal interpretations. Although FreeSurfer is a commonly used protocol for hippocampal segmentation, automated delineation of hippocampal subfields is a challenging task in neuroimaging research due to their small size, measurement error due to neuroanatomical complexity (e.g., ambiguity in identifying adjacent subfield boundaries and segmentation errors), and variability in automated and/or manual segmentation protocols (Canada et al., 2024; Schmidt et al., 2018; Yushkevich et al., 2015). Limitations of MRI, such as increased like-lihood of signal loss in medial temporal lobes of the brain due to susceptibility-induced magnetic field gradients, may also differentially impact subfield measurements (Olman et al., 2009). Examining differential relationships between constituent tests of the episodic memory composite score and hippocampal subfield volumes was limited by data availability and the lack of additional memory tasks (e.g., visual episodic memory tests or pattern separation tasks). Although longitudinal data were available in ADNI, examination of 36-month follow-up revealed a 56% attrition rate from baseline (*n* = 586) to 36-month follow-up (*n* = 262), as well as differential rates of attrition based on disease severity at baseline (A+T+N+ = 69% attrition; A-T-N- = 52% attrition). Given that attrition bias, particularly selective attrition, can threaten internal and external validity (Boykin et al., 2016; Miller & Wright, 1995), longitudinal data were not considered in the current study.

Despite these limitations, this study had several strengths, including the examination of domain-specific cognitive function, episodic memory, and its relationship with CSF GAP-43, total hippocampal volume, and hippocampal subfield volumes. Testing differential biomarker associations with episodic memory is essential given that impairments in specific cognitive domains have differing consequences for AD risk (Bradfield et al., 2018; Espinosa et al., 2013; Yaffe et al., 2006). Importantly, our analyses controlled for ATN status to account for variability in episodic memory performance explained by hallmark AD biomarkers. Use of a composite score allowed for a robust measure of episodic memory performance without reliance on a single test item (Brooks et al., 2011; Crane et al., 2012). We also examined total and subfield hippocampal volume as potential moderators of the relationship between CSF GAP-43 and episodic memory performance, allowing for comparisons between an aggregate measure and regional variability within the hippocampus.

In conclusion, the current study demonstrated that CSF GAP-43 was associated with episodic memory performance independent of robust biomarkers of AD, such as ATN status. The negative association between CSF GAP-43 and episodic memory performance was stronger in older adults with average or below-average hippocampal volume. These findings suggest that CSF GAP-43 may serve as a biomarker correlate of episodic memory performance, an early hallmark clinical symptom of AD, in older adults with reduced hippocampal structural integrity.

## Supplementary Material

Supp 1

Supplemental data for this article can be accessed online at https://doi.org/10.1080/13825585.2025.2562203.

## Figures and Tables

**Table 1.** Demographic and study characteristics of the sample. Values represent the mean (standard deviation) unless indicated otherwise.

Independent Variable	Overall (N = 586)
Age (years)	72.6 (±7.2)
Education (years)	16.1 (±2.7)
Sex, n (% female)	268 (46%)
*APOE*-ɛ4, n (% carrier)	268 (46%)
Mini-Mental State Examination	27.7 (±2.5)
Episodic memory composite (z-score)	0.41 (±0.9)
CSF GAP-43 (pg/mL)	5270.0 (±2851.1)
Total hippocampal volume (cm^3^)	7027.6 (±1108.9)
Subiculum volume (cm^3^)	552.7 (±89.2)
Presubiculum volume (cm^3^)	401.9 (±72.8)
Dentate gyrus volume (cm^3^)	504.9 (±74.9)
CA1 volume (cm^3^)	318.6 (±41.4)

Abbreviations: AD = Alzheimer’s disease; CA1 = Cornu Ammonis-1; CSF = cerebrospinal fluid; GAP-43 = growth-associated protein 43; SNAP = suspected non-Alzheimer pathophysiology.

**Table 2.** Step 4 of each hierarchical linear regression with CSF GAP-43 x Hippocampal ROI interactions on episodic memory performance. A significant interaction between GAP-43 and total hippocampal volume and hippocampal subfield volume (subiculum, presubiculum, and dentate gyrus) on episodic memory performance was observed.

Composite Episodic Memory (z-score)										
	Total hippocampal volume		Subiculum volume		Presubiculum volume		Dentate gyrus volume		CA1volume	
Independent Variable	$\hat{\beta }$	t	$\hat{\beta }$	t	$\hat{\beta }$	t	$\hat{\beta }$	t	$\hat{\beta }$	t
Age	−0.02	−0.62	−0.03	−1.02	−0.01	−0.37	−0.06^*^	−2.03	−0.13^***^	−4.21
Education	0.17^***^	6.01	0.17^***^	5.87	0.17^***^	6.31	0.18^***^	6.10	0.17^***^	5.52
Sex (female)	0.23^***^	4.08	0.25^***^	4.30	0.25^***^	4.50	0.29^***^	5.00	0.33^***^	5.34
*APOE*-ɛ4 status	−0.16^*^	−2.55	−0.15^*^	−2.32	−0.14^*^	−2.19	−0.18^**^	−2.75	−0.23^**^	−3.31
ATN SNAP group	−0.01	−0.17	−0.01	−0.13	−0.02	−0.25	−0.02	−0.23	−0.02	−0.18
ATN AD group	−0.46^***^	−6.36	−0.45^***^	−6.20	−0.46^***^	−6.47	−0.51^***^	−6.95	−0.58^***^	−7.61
CSF GAP-43	−0.09^**^	−3.23	−0.08^**^	−2.79	−0.09^**^	−3.18	−0.09^**^	−3.15	−0.08^**^	−2.70
Hippocampal ROI	0.35^***^	10.88	0.33^***^	10.07	0.36^***^	11.33	0.27^***^	8.78	0.13^***^	4.16
**GAP-43 × Hippocampal ROI**	0.06*	2.09	0.06^*^	1.98	0.06^*^	2.06	0.06*	2.07	0.04	1.28
**R** ^**2**^	0.46		0.45		0.47		0.43		0.37	
△**R**^**2**^	0.004^*^		0.004^*^		0.004^*^		0.004^*^		0.002	
**Model F**	*F*(9,576) = 55.23^***^		*F*(9,576) = 52.25^***^		*F*(9,576) = 57.17^***^		*F*(9,576) = 47.71^***^		*F*(9,576) = 37.30^***^	

* = *p* < 0.05

** *p* < 0.01

*** *p* < 0.001.

Abbreviations: AD = Alzheimer’s disease; ATN = amyloid-β/tau/neurodegeneration; CA1 = Cornu Ammonis-1; CSF = cerebrospinal fluid; GAP-43 = growth-associated protein 43; ROI = region-of-interest; SNAP = suspected non-Alzheimer pathophysiology.

## Data Availability

The data supporting the findings of this study are openly available in the Alzheimer’s Disease Neuroimaging Initiative database, https://adni.loni.usc.edu/. Codes generated and used in this work are available in a data repository, https://github.com/osubbal/GAP43-HC-Memory_ALee.
