# Supplementary material for: Hippocampal volume moderates the association between cerebrospinal fluid growth-associated protein 43 and episodic memory performance in older adults
Source: Neuropsychol Dev Cogn B Aging Neuropsychol Cogn. Author manuscript; Available in PMC 2025 Dec 17. (PMC12710805; doi:10.1080/13825585.2025.2562203)
Supplement: Supp 1 [file NIHMS2113471-supplement-Supp_1.docx]

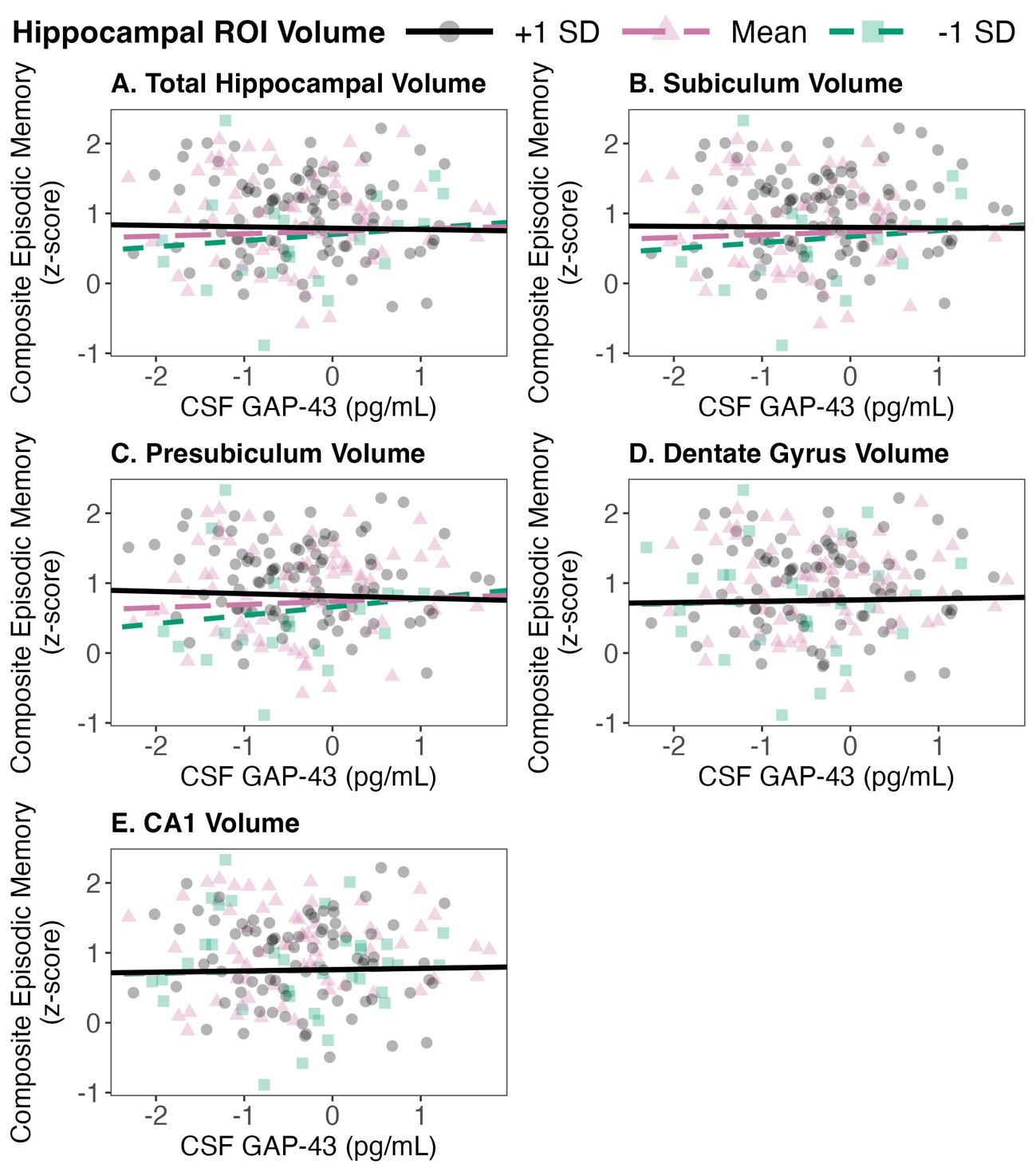


**Supplementary Figure 1. Associations between CSF GAP-43 and episodic memory performance by hippocampal volume in the ATN no AD pathology group (n = 182).** There were no main effects of CSF GAP-43 and hippocampal ROIs (apart from presubiculum volume, *p* < 0.05) or GAP-43 x hippocampal ROI interactions observed.

Abbreviations: ATN = amyloid-β/tau/neurodegeneration; CA1 = Cornu Ammonis-1; CSF = cerebrospinal fluid; GAP-43 = growth-associated protein 43; ROI = region-of-interest; SD = standard deviation.


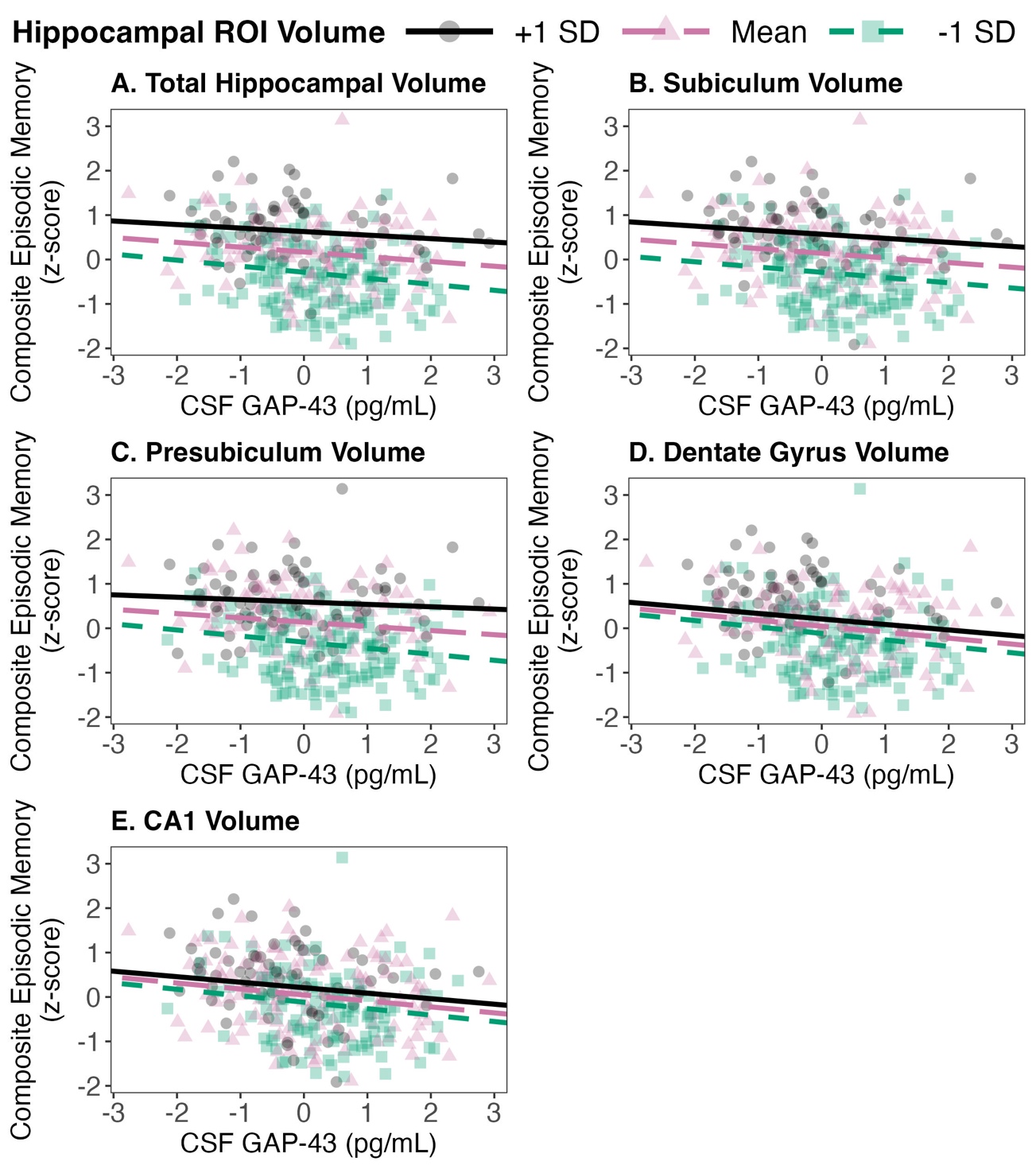


**Supplementary Figure 2. Associations between CSF GAP-43 and episodic memory performance by hippocampal volume in the ATN AD pathology group (n = 312).** There were main effects of CSF GAP-43 ($\hat{\beta}$ = -0.16, *p* < 0.001) and hippocampal ROIs ($\hat{\beta}$s = 0.17 - 0.46, *p*s < 0.001) on episodic memory performance. However, there were no GAP-43 x hippocampal ROI interactions observed.

Abbreviations: ATN = amyloid-β/tau/neurodegeneration; CA1 = Cornu Ammonis-1; CSF = cerebrospinal fluid; GAP-43 = growth-associated protein 43; ROI = region-of-interest; SD = standard deviation.
